# Pseudo-Exacerbation in Primary Progressive Multiple Sclerosis: A Case Report

**DOI:** 10.7759/cureus.117167

**Published:** 2026-09-29

**Authors:** Parisa Moshfeqa, Norhan Shaykhon, Sooraj Viswanathan, Gavin Ross

**Affiliations:** 1 Internal Medicine, Glangwili General Hospital, Carmarthen, GBR; 2 Psychiatry, Withybush General Hospital, Pembrokeshire, GBR; 3 Internal Medicine, Withybush General Hospital, Pembrokeshire, GBR; 4 Gastroenterology, Withybush General Hospital, Pembrokeshire, GBR

**Keywords:** autoimmune neurology, demyelinating disorders, primary progressive multiple sclerosis, pseudo-exacerbation, systemic autoimmune disease

## Abstract

Primary progressive multiple sclerosis (PPMS) is characterised by gradual neurological deterioration from disease onset without distinct relapses. Acute neurological worsening in patients with PPMS can present a diagnostic challenge, particularly when occurring in the context of systemic infection, where pseudo-relapse may mimic disease progression or a true inflammatory relapse. We report the case of a 65-year-old woman with established PPMS who presented with fever, sore throat, and acute bilateral lower-limb weakness. Assessment included a detailed history, neurological examination, and laboratory investigations demonstrating increased inflammatory markers. Neurology review was undertaken to distinguish pseudo-relapse from true relapse. In the absence of new focal neurological deficits and with a probable infective trigger, the episode was considered a pseudo-relapse secondary to presumed pharyngitis. The patient was treated with empirical antibiotics and supportive rehabilitation without corticosteroids. Significant clinical improvement was observed, with return to baseline mobility and functional status. As no contemporaneous MRI was performed, the diagnosis and treatment were based on clinical grounds. At the two-month follow-up, she remained clinically stable with no new neurological deficits. This case highlights the importance of distinguishing pseudo-relapse from true relapse in patients with PPMS presenting with acute neurological deterioration. Careful clinical assessment and recognition of reversible systemic triggers can help avoid unnecessary corticosteroid exposure while ensuring appropriate management of the underlying cause.

## Introduction

Multiple sclerosis (MS) is a chronic immune-mediated disease of the central nervous system characterised by inflammation, demyelination, axonal injury, and neurodegeneration [1,2]. Most patients initially follow a relapsing-remitting disease course, whereas approximately 10-15% present with primary progressive multiple sclerosis (PPMS), a phenotype characterised by gradual accumulation of neurological disability from disease onset without clearly defined relapses and remissions [1]. Compared with relapsing-remitting MS, PPMS commonly presents with progressive gait impairment and lower-limb weakness, reflecting a greater burden of spinal cord involvement and neurodegenerative pathology [1,2].

Relapses are defined as episodes of new or worsening neurological symptoms lasting at least 24 hours in the absence of fever, infection, or another alternative explanation [3]. In contrast, a pseudo-relapse refers to the temporary worsening of pre-existing neurological deficits precipitated by systemic stressors such as infection, pyrexia, heat exposure, or metabolic disturbance [3-6]. These episodes do not represent new inflammatory demyelinating activity and are thought to result from transient impairment of conduction within previously demyelinated neural pathways [4,7]. Published cases documented symptom exacerbation during systemic infection [8], and subsequent studies described fever‑related conduction block and pseudo‑relapse physiology in MS [9,10].

Distinguishing a true relapse from a pseudo-relapse is clinically important because management differs substantially. While true relapses may warrant further investigation and treatment with corticosteroids, pseudo-relapses are managed by identifying and addressing the underlying trigger [3,5]. Accurate diagnosis helps avoid unnecessary corticosteroid exposure and ensures timely treatment of reversible causes of neurological deterioration.

Although pseudo-exacerbations are well recognised in MS, distinguishing them from disease progression or superimposed inflammatory activity may be challenging in patients with PPMS who present with acute neurological deterioration. We report a patient with long-standing PPMS who developed profound lower-limb weakness during an episode of febrile illness and discuss the clinical reasoning with suggestive and previously documented triggers in the literature to avoid unnecessary corticosteroids or inappropriate escalation of disease-modifying therapy [7,11].

## Case presentation

A 65-year-old female with a long-standing, clinically confirmed diagnosis of PPMS, first diagnosed in 2008 (16 years before presentation), presented to the hospital with a one-day history of sore throat and worsening bilateral lower limb weakness. Her baseline neurological disability was significant, with a most recent Expanded Disability Status Scale (EDSS) score of 6.5 [12], recorded three months before admission, and a Multiple Sclerosis Walking Scale (MSWS-12) score of 47/60 [13]. At baseline, she was able to mobilise approximately 10 m using a Zimmer frame. She was not receiving any disease-modifying therapy at the time of presentation, although a trial of fampridine was being considered to improve mobility. Her medical history was notable for four urinary tract infections during the preceding year. On the day of admission, she was unable to get out of bed because of profound bilateral leg weakness.

Upon arrival, she was haemodynamically stable; however, an elevated temperature of 38.7°C was noted. Apart from a sore throat, she denied any other symptoms suggestive of an upper respiratory tract infection. She also denied any gastrointestinal, urinary, or visual disturbances. Oral examination revealed erythematous mucosa and tonsils; however, there was no pus on the tonsils.

On admission, neurological examination revealed power of 1/5 on hip, knee, and ankle flexion and extension in the left lower limb; 1/5 on hip flexion and extension in the right lower limb; and 5/5 in both upper limbs. Deep tendon reflexes were brisk bilaterally, and plantar responses were flexor bilaterally. There was no sensory deficit in the upper or lower limbs. Functionally, she was unable to get out of bed, compared with her baseline of walking 10 m with a Zimmer frame. Higher cortical function, cranial nerves, cerebellar function, speech, and vision were normal.

Blood tests demonstrated raised inflammatory markers, including leukocytosis and an elevated C-reactive protein. The full laboratory results on admission are summarised in Table 1.

**Table 1:** Laboratory investigations on admission. Total calcium and estimated glomerular filtration rate were reported without an accompanying reference range.

Parameter	Patient value	Reference range
Full blood count		
White blood cell count	16.7 × 10⁹/L H	4.0–11.0
Haemoglobin	147 g/L	115–165
Platelet count	197 × 10⁹/L	150–400
Red blood cell count	4.82 × 10¹²/L	3.80–5.50
Haematocrit	0.46 L/L	0.37–0.47
Mean cell volume	96 fL	80–100
Mean cell haemoglobin	30.6 pg	27.0–33.0
Red cell distribution width	12.6%	11.0–14.8
Neutrophil count	15.5 × 10⁹/L H	1.7–7.5
Lymphocyte count	0.6 × 10⁹/L L	1.0–4.5
Monocyte count	0.5 × 10⁹/L	0.2–0.8
Eosinophil count	0.0 × 10⁹/L	0.0–0.4
Basophil count	0.0 × 10⁹/L	0.0–0.1
Inflammatory marker		
C-reactive protein	29 mg/L H	<5
Bone profile		
Calcium	2.45 mmol/L	—
Calcium (adjusted to albumin)	2.40 mmol/L	2.20–2.60
Liver function tests		
Bilirubin	16 µmol/L	<21
Total protein	73 g/L	60–80
Albumin	48 g/L	35–50
Alkaline phosphatase	109 U/L	30–130
Alanine transaminase	19 U/L	<33
Renal profile and electrolytes		
Sodium	142 mmol/L	133–146
Potassium	4.1 mmol/L	3.5–5.3
Urea	3.5 mmol/L	2.5–7.8
Creatinine	56 µmol/L	46–92
Estimated glomerular filtration rate	>90 mL/minute/1.73m²	—
Other investigations		
Random glucose (serum)	6.6 mmol/L	3.0–7.7
D-dimer (quantitative)	594 µg/L H	<500
Blood culture (venous)	Negative	—
Viral respiratory swab	Negative	—

Based on the clinical findings, the initial differential diagnosis included a pseudo-relapse secondary to infection, true MS relapse involving the spinal cord, metabolic derangements, medication-related weakness, deconditioning, and other neurological or systemic causes of acute weakness. Neurology was consulted and considered a pseudo-relapse of MS secondary to infection to be the most likely diagnosis. A true MS relapse was considered less likely because there were no new sensory symptoms, visual changes, sphincter dysfunction, cerebellar signs, or other focal neurological deficits suggestive of new inflammatory demyelinating activity. Metabolic causes were excluded through routine laboratory investigations, which revealed no clinically significant electrolyte abnormalities or other metabolic disturbances likely to account for her presentation. Medication-related weakness was also considered unlikely, as she was not receiving disease-modifying therapy, had not recently received corticosteroids, and was taking no medications known to precipitate acute neuromuscular weakness. Although deconditioning may have contributed to her functional impairment, the abrupt onset of severe weakness over a single day, coinciding with fever and pharyngitis, was not typical of progressive deconditioning. Other neurological and systemic causes, including stroke, peripheral neuropathy, compressive myelopathy, and sepsis-related weakness, were considered less likely given the symmetrical distribution of weakness, preserved sensation, absence of cranial nerve or upper limb involvement, stable haemodynamic status, and clear temporal relationship to the febrile illness.

The management plan focused on treatment of the possible infective illness while monitoring for evolving neurological deficits and bladder or bowel dysfunction. MRI of the brain and spinal cord was considered but deferred because of her early neurological improvement. Neuroimaging would have been pursued had there been no demonstrable recovery in lower limb function to exclude a true MS relapse or alternative spinal pathology. Steroids were not administered because the clinical picture was felt to be most consistent with a pseudo-relapse rather than an acute inflammatory relapse.

Her established diagnosis of PPMS was supported by previous MRI. The most recent MRI (Figure 1), performed one year before admission, demonstrated several demyelinating lesions consistent with MS but showed no evidence of active disease. No repeat neuroimaging was performed during this admission.

**Figure 1:**
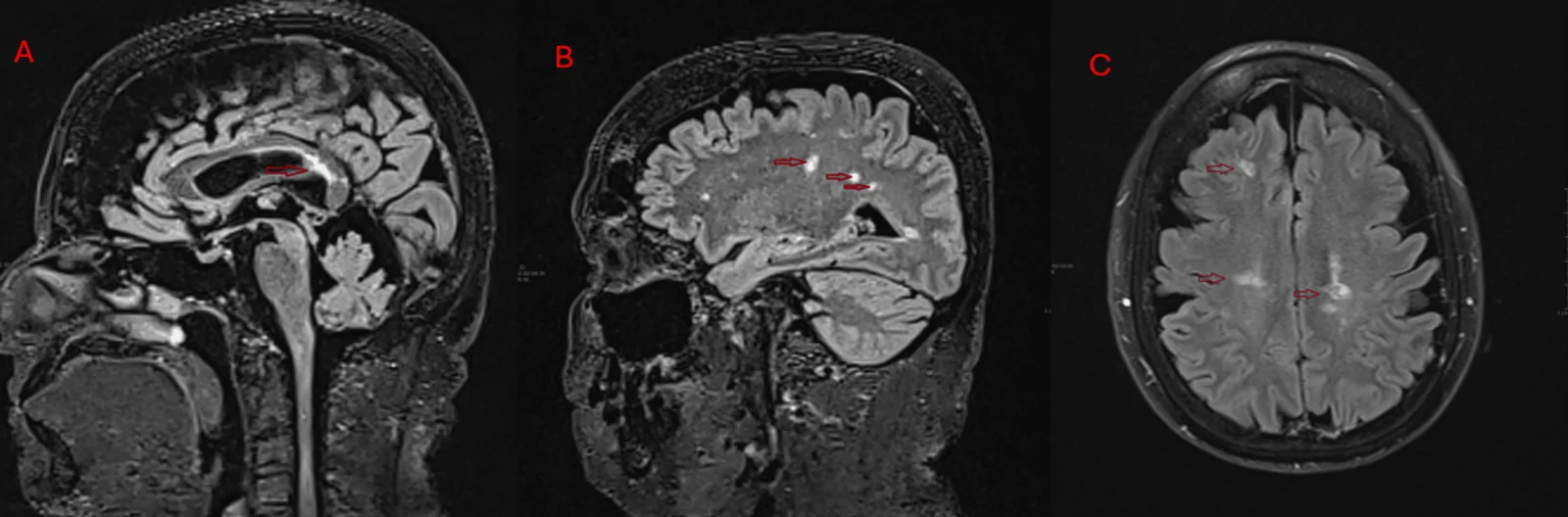
Brain magnetic resonance imaging demonstrating demyelinating lesions. Previous brain magnetic resonance imaging (FLAIR) demonstrating multiple T2/FLAIR-hyperintense lesions in a distribution typical of demyelinating plaques. A: Sagittal FLAIR image; the arrow indicates a hyperintense lesion involving the corpus callosum (callososeptal interface). B: Parasagittal FLAIR image showing juxtacortical and subcortical white matter lesions (arrows). C: Axial FLAIR image showing periventricular and subcortical white matter lesions (arrows). Post-contrast sequences (not shown) demonstrated no abnormal enhancement, arguing against active disease. The images were acquired before the index admission; repeat neuroimaging was not performed during this presentation. FLAIR: fluid-attenuated inversion recovery

Her neurological recovery followed a clear day‑by‑day trajectory. On day one, she remained profoundly weak with bilateral lower‑limb power of 1/5. By day two, strength had improved to 1/5 in the left leg and 3/5 in the right leg. Continued improvement was observed by day six, when lower‑limb power reached 3/5 (left) and 4/5 (right), enabling her to mobilise with a Zimmer frame with assistance, close to her baseline level of independent Zimmer‑frame mobilisation by day six. However, the rehabilitation team advised that she required further stair training before returning home. She was therefore transferred to a rehabilitation unit, where she completed stair-climbing practice and was discharged the following week.

At follow‑up in the neuroinflammatory clinic two months later, assessment revealed an EDSS of 6, a return to her baseline functional status, and no new neurological deficits [12]. The return to functional baseline without corticosteroid therapy further supported a suggestive infectious trigger for a pseudo-relapse.

## Discussion

MS encompasses several clinical courses, including relapsing‑remitting, primary progressive, and secondary progressive disease, with relapsing‑remitting MS being the most common phenotype [6]. PPMS is characterised by steady neurological decline from onset, typically with infrequent plateaus and only minor transient improvements, and it presents at a later age compared with relapsing‑remitting MS. Alroughani et al. identified several predictors of accelerated disease progression, including onset after age 40, male sex, three or more relapses, and spinal cord symptoms at presentation, highlighting the prognostic significance of early cord involvement [14].

PPMS most commonly presents with progressive spastic paraparesis, reflecting chronic corticospinal tract involvement rather than complete paraplegia [1]. This distinction is clinically important, as paraparesis denotes gradually worsening lower‑limb weakness and spasticity rather than the profound motor loss implied by paraplegia. In keeping with this phenotype, our patient first presented at age 49 with bilateral leg weakness, consistent with spinal cord-predominant PPMS.

Acute inflammatory relapses in MS are defined as new or worsening neurological symptoms lasting more than 24 hours in the absence of fever or infection [3]. In this case, the patient’s deterioration occurred in the context of presumed pharyngitis and pyrexia, and an absence of new neurological deficits did not meet the definition of a relapse. This pattern is more consistent with a pseudo-exacerbation, in which temperature-related impairment of axonal conduction temporarily worsens pre-existing deficits, with symptoms returning to baseline once the trigger resolves [4].

She was treated empirically with penicillin for presumed pharyngitis based on fever and tonsillar erythema. No throat culture or rapid streptococcal antigen test was performed, and blood cultures and a viral respiratory swab were negative; a bacterial or streptococcal aetiology was therefore not confirmed. However, the presence of tonsillar erythema, fever, and clinical response to antibiotics supported this working diagnosis. Corticosteroids were avoided as they were not clinically indicated [5]. The decision to avoid corticosteroids was based on structured clinical reasoning rather than an assumption that this was not a true relapse. Several features supported pseudo‑exacerbation: the presence of fever and presumed infective trigger, absence of new focal neurological deficits, intact sensory and cranial nerve examination, and a pattern of weakness consistent with her baseline PPMS phenotype. Importantly, her lower‑limb strength improved promptly after the febrile illness resolved, which is aligned with pseudo‑exacerbation rather than new inflammatory disease activity. In this context, the risks of unnecessary corticosteroid exposure were judged to outweigh potential benefit, and neurology advised clinical monitoring rather than corticosteroid therapy.

Infection-triggered pseudo-exacerbations are more frequently reported in relapsing‑remitting MS; therefore, this case is notable in demonstrating a pseudo‑exacerbation in a patient with PPMS [6,7]. The underlying mechanism, i.e., transient conduction block in demyelinated axons precipitated by systemic stressors, is shared across multiple sclerosis phenotypes [4]. Their clinical recognition in PPMS is more challenging because acute worsening is often attributed to irreversible progression rather than reversible physiological triggers. The educational value of this case therefore lies in illustrating the diagnostic uncertainty.

Differentiating true relapse from pseudo‑relapse is primarily clinical, commonly triggered by systemic stressors such as infection, pyrexia, and metabolic disturbances, typically resolving with treatment and not requiring repeat MRI when the presentation is characteristic and neurological improvement is observed [3,6,10,11]. A thorough history and examination are essential to identify potential triggers, and neuroimaging may be used selectively. In this case, repeat MRI was not performed because the patient improved clinically once the systemic illness resolved. We acknowledge this as a limitation, as no further inflammatory activity can be excluded. Therefore, our diagnosis remains a clinical conclusion supported by the presence of pyrexia, lack of new neurological deficits, and improved functionality without corticosteroid therapy.

Rehabilitation remains an integral component of MS management. During pseudo‑relapses, timely physiotherapy input can facilitate return to baseline function [5]. In our case, the patient received physiotherapy support during admission and following discharge, which contributed to her recovery.

Accurate identification of pseudo‑exacerbation is crucial to avoid unnecessary corticosteroid administration and inappropriate modification of immunosuppressive therapy, particularly when symptoms are not disabling [6]. Further research is needed to refine guidance on managing MS patients with concurrent infection and to better understand the balance between immunosuppression, infection risk, and functional outcomes.

## Conclusions

This case highlights that a pseudo-exacerbation in PPMS may present with abrupt, clinically significant worsening that mimics progression or true inflammatory relapse. Recognising reversible deterioration requires careful assessment of systemic triggers, exclusion of alternative causes of acute weakness, and multidisciplinary input. The patient’s improvement following the resolution of presumed infective pharyngitis supported a pseudo‑relapse, although the absence of contemporaneous MRI means new inflammatory activity cannot be excluded definitively. This case underscores the importance of structured diagnostic reasoning, cautious use of corticosteroids, and early rehabilitation when evaluating sudden deterioration in PPMS.
